# The role of healthcare providers in sustainable return-to-work for individuals with common mental disorders

**DOI:** 10.1093/bmb/ldaf018

**Published:** 2025-10-14

**Authors:** Zoe Can, Cristian A Vasquez, Susan E Peters, Jeremy F Dawson

**Affiliations:** Sheffield University Management School, The University of Sheffield, Conduit Road, Sheffield S10 1FL, United Kingdom; Sheffield University Management School, The University of Sheffield, Conduit Road, Sheffield S10 1FL, United Kingdom; Department of Social and Behavioral Sciences, Harvard T.H. Chan School of Public Health, 677 Huntington Avenue, Boston 02115, United States; Sheffield University Management School, The University of Sheffield, Conduit Road, Sheffield S10 1FL, United Kingdom

**Keywords:** healthcare, mental health, organizational psychology, sustainable return-to-work

## Abstract

**Background:**

Many individuals with common mental disorders (CMDs) face challenges after returning to work following sickness absence. Healthcare providers and healthcare systems are well-placed to provide returnees with support which can facilitate sustainable return-to-work.

**Sources of data:**

This narrative review has integrated data and literature from journal articles, reports, book chapters, and official NHS and UK statistics bodies.

**Areas of agreement:**

Individual healthcare providers and healthcare systems can take various actions to support sustainable return-to-work for individuals with CMDs. These include utilizing shared decision-making, maintaining communication with other stakeholders, and delivering suitable interventions. Healthcare leadership systems should prioritize improving relevant provider knowledge and skills as many lack confidence managing cases involving return-to-work.

**Areas of controversy:**

Healthcare providers face uncertainty about which supportive actions fall within their scope of practice.

Growing points: Research interest in sustainable return-to-work appears to have grown in recent years.

**Areas timely for developing research:**

Future research should seek to clarify the definition of sustainable return-to-work and examine how resources across system levels can support sustainable return-to-work.

## Introduction

Common mental disorders (CMDs), such as anxiety and depression, are one of the leading causes of workplace sickness absence in developed nations [1]. The scale of this challenge is evident in the United Kingdom: mental health conditions accounted for over 18 million lost working days in 2022 [2], and a study of 68 general practices identified that mental disorders comprised over 32% of general practioner (GP) issued fit notes [3]. The economic impact is also substantial, with UK employers facing costs exceeding £7 billion in 2023, a 19% increase from 2020/2021 [4]. Despite these challenges, research indicates that most individuals experiencing mental health conditions do want to remain in meaningful employment [5]. Being employed and the quality of work is an important social determinant of health [6]. Work provides not only economic stability but also has the potential to contribute to health and wellbeing, for example, by providing a sense of meaning, purpose, routine, and social connection [7–9]. Therefore, facilitating return-to-work (RTW) after mental health absences can help individuals access these benefits whilst avoiding the potential negative consequences of long-term sickness absence [9].

RTW is often thought of as a binary outcome—has the worker returned to work or not—however, in reality, it is much more complex and dynamic, and is better conceptualized as a process. That is, RTW can encapsulate both the outcome of returning to work following an absence, and the process by which someone arrives at that point and navigates what follows after their return [10]. In recent years, the concept of sustainable RTW (SRTW) has gained greater research interest, acknowledging the need to consider longer-term RTW outcomes and experiences following a worker’s initial resumption of work after ill-health [11]. SRTW considers what occurs after the first re-entry to work, and recognizes that an initial RTW does not provide the complete picture; what happens in the period after this first return, when the worker is likely most vulnerable, and beyond, can convey a more accurate depiction of the actual RTW outcome. SRTW itself does not currently have one agreed-upon definition. Nielsen *et al*. [11] proposed a preliminary definition, suggesting that SRTW may be characterized by returning to contracted hours with equal pay, having minimal absence recurrence, displaying good work functioning, and not dropping out of the workforce.

Research indicates that many individuals with CMDs face challenges after initial RTW following a mental health-related absence. In an ideal situation, a returnee would be able to maintain their employment and thrive from their work after their initial return, but unfortunately, many returnees do not have this straightforward and positive experience. A study examining fit notes in the UK found 18% of individuals with a mental health-related absence had a recurrent absence also due to their mental health [12]. This same study found that, compared to absences caused by other conditions, mental health-related absences had the highest amount of recurrence. A recent study from the Netherlands had similar findings, with 16% of those with a long-term mental health-related absence having a recurrent long-term absence in the 3 years following their initial RTW [13]. This literature suggests that many returnees encounter difficulties post-return. Understanding and addressing challenges, and identifying beneficial support, may be helpful for enabling returnees to achieve SRTW. Healthcare providers are a main source of support for returnees, who may be able to positively impact the returnee experience. The current narrative review explores the role of healthcare providers in supporting the SRTW process for their patients with CMDs, identifying key opportunities for intervention and highlighting how they can utilize their unique position to help returnees.

Nielsen *et al.* developed a framework that allows for the classification of resources at five levels which may support SRTW: individual (I), group (G), leader (L), organization (O), and overarching context (O) [11], referred to as the IGLOO model, as shown in Table 1. Healthcare providers play a significant role in the RTW process as actors within the IGLOO model. The framework provides examples of resources, such as job crafting at the individual level, family at the group level, line manager behaviour at the leader level, job design at the organization level, and country legislation at the overarching context level. Work and non-work contexts are both considered within this model. Nielsen *et al*. [11] propose that insufficient resources at the five levels may lead to undesirable outcomes such as absence recurrence, whilst greater resource levels may be beneficial for SRTW. In this model, healthcare providers, such as GPs, psychiatrists, and psychologists, are identified as a potential source of leader-level resources, whilst occupational health services are recognized at the organization level. Certain allied health professionals may also play a key role in RTW [14]. Altogether, healthcare providers are in a prime position to facilitate and support SRTW for those with CMDs.

**Table 1:** IGLOO model levels^11^ with example healthcare-related resources.

**IGLOO model level**	**Potential resource(s)**
Individual	Patient engagement and motivation
Group	Family support whilst individual is utilizing healthcare services
Leader	Support and treatment from healthcare providers such as GPs, psychiatrists, psychologists, and allied health professionals
Organization	Occupational health service provisions
Overarching context	Economic climate; national legislation; structure of healthcare system

A necessary first step is for healthcare providers to recognize themselves as a valuable source of support for returnees, especially after the initial resumption of work. Encouragingly, previous research has found that 90% of GPs somewhat or completely agree that helping patients to RTW or stay in work is an important part of their role [15]. This study also found that 76% of surveyed GPs agreed that a patient returning to work or staying in work was an important indicator of clinical success. To enable healthcare providers to translate their views into action, it is essential to identify specific strategies which allow healthcare providers to support SRTW within their scope of practice.

## Organizing RTW timeframes and making decisions

There are various ways healthcare providers may be able to support the SRTW process. First, they can be involved in organizing and establishing suitable RTW schedules for their patients, for example, through the provision of fit notes. Through focus group discussions with individuals with experience of a CMD-related absence, Nybergh *et al*. [16] found participants were thankful for healthcare providers suggesting longer absence lengths or slowing the RTW down when necessary. Crucially, decisions on RTW timing must attempt to reconcile two issues: RTWs which are too fast appear to be detrimental, but longer absences are associated with exiting the workforce altogether [17]. Healthcare providers must therefore take an approach which provides individuals with adequate rest and recovery time, whilst encouraging and supporting appropriate RTW when the individual appears capable of some level of work. Progressive returns, also known as gradual or phased returns, offer one option for steadily re-introducing individuals to work by enabling returnees to build up working hours over time. In a study of individuals returning to work following a depression-related absence, it was identified that a lack of progressive RTW may partly explain the observed relapses which occurred between 1 month and 3 months after the initial RTW [18]. However, progressive returns are only effective if work demands are also increased gradually [19]. Whilst healthcare providers cannot control a patient’s work demands, they can take them into consideration when planning an RTW: the patient’s health and working capacity should be balanced alongside the job’s design to formulate a realistic RTW schedule.

Research has also indicated that the processes by which RTW decisions are made are influential. Corbière *et al*. [18] discovered that a disagreement between the healthcare provider and the returnee about RTW timing may contribute to future instances of relapse. Interestingly, the observed discrepancies did not solely relate to the healthcare provider suggesting a timeframe that the returnee thought was too fast; some discrepancies stemmed from the returnee wanting to return sooner than the healthcare provider recommendation. Based on this, the study authors emphasized the importance of basing scheduling plans on shared decision-making between practitioner and returnee. Another study examined the differences between the views of occupational physicians and returnees on which factors were important for preventing relapse [20]. This research found that workers rated sufficient absence time before the RTW as more important for SRTW than occupational physicians. Differences of opinion may also exist on other aspects of an RTW, beyond timing. The same study found workers rated better financial compensation as more important for SRTW than occupational physicians, whilst occupational physicians rated a wide range of support from contacts as more important than workers. Ultimately, the views of healthcare providers and patients may not always align, and discrepancies can often exist. Healthcare providers should recognize this and place value on the worker’s voice, utilizing shared decision-making. Emphasizing this, in a recent study of 20 workers who had returned to work following a CMD-related absence, participants noted that shared decision-making, and having their own opinions on their health and recovery considered by GPs, mental health professionals, and occupational therapists, was important to them [21]. Additionally, in a longitudinal study involving monthly interviews with returnees with CMDs, one identified facilitator of SRTW was healthcare taking an individualized approach, with treatment well-suited for the individual’s specific needs [22]. Altogether, healthcare providers may be able to support SRTW outcomes by taking a personalized approach and ensuring that the voice of each individual returnee is heard during decision-making processes. However, a recent review noted that healthcare providers can encounter challenges when trying to utilize shared decision-making in mental health-related care, including patient symptoms reducing decision-making abilities, and patients feeling they cannot be fully honest with providers due to stigma [23]. The review concluded that some obstacles are surmountable whilst others may be inherent to the provision of mental health care, but that the overall aim should be to make care as shared as possible. Accordingly, individual healthcare providers should operate with this aim in mind when navigating RTW cases.

## Coordinating with stakeholders and boosting other resources

As noted earlier, healthcare providers are recognized as a potential source of resources in the IGLOO model [11]. However, healthcare providers may also be in a suitable position to boost other resources within the model. For example, in the UK, doctors, nurses, occupational therapists, pharmacists, and physiotherapists can issue fit notes which indicate whether the patient’s health status renders them potentially capable of work or not, with a section where providers can write suggestions for work accommodations, such as amended duties [24]. However, recent data from NHS England [25] showed that, from March 2023 to April 2024, 70 972 fit notes were issued as ‘may be fit for work’ without any advice provided on the form. When healthcare providers have knowledge of an individual’s work context, they should ensure fit notes provide guidance on workplace accommodations to ensure workers and employers are well advised. To allow for this, when possible, consultations with patients seeking fit notes should contain some discussion of the work context. Accommodations, such as modifying job duties, reducing overtime, reducing travel, or pairing returnees with a colleague, can be implemented by the workplace to support SRTW [14, 18]. In such circumstances, healthcare providers may be able to boost other resources within the IGLOO model to facilitate SRTW. This brings to light the collaborative effort which can be required to achieve SRTW: healthcare providers can propose workplace adaptations, but they rely on the workplace implementing suggested changes. Another example of this is that healthcare providers can offer treatments for CMDs, but the individual is required to engage with the support offered. Ultimately, healthcare providers should therefore seek opportunities to coordinate and communicate with relevant individuals and groups. Emphasizing the value of coordination, a study of returnees, managers and occupational physicians found adequate coordination between the clinician, occupational physician, supervisor and employee to be one of the most important influences on RTW [26]. Other research shows stakeholder coordination to be a helpful but underutilized practice, with Holmlund *et al*. [27] finding through interviews with managers that few experienced support from primary healthcare to help them identify returnee needs, with this identified as a barrier to RTW. Whilst opportunities for collaboration may be inherently limited by the resources available to healthcare providers and patient confidentiality responsibilities, in scenarios where providers have knowledge of an individual’s work context, they should ensure fit notes are thorough, specific and tailored, with this presenting an avenue for boosting communication between healthcare and employers. Every workplace is a unique environment, but current UK guidance states that healthcare provider assessments are not required to be job-specific [28]. However, providing tailored evaluations and guidance could enhance communication between employers and healthcare and ensure that workplaces have clear and actionable directions. Therefore, when possible, healthcare providers should provide detailed fit notes which take the specific work someone is returning to into account.

As discussed, resources within the IGLOO model [11] may influence other resources. The fifth level, the overarching context level, can play a key role, affecting the support healthcare providers can offer. If the broader social, political, and economic climate hinders the support a healthcare provider can give, this places a clear constraint on the positive impact a provider can have. Conversely, a supportive context can allow providers to deliver improved patient support. For example, when providers have adequate time for consultations, discussing a patient’s work circumstances may be more feasible. Individual healthcare providers may be able to positively impact certain contextual factors at the overarching context level, for example, by contributing to the reduction of the societal stigma surrounding mental health problems. A systematic review found that mental health stigma is common amongst certain healthcare providers [29], and so reducing any personal stigma providers hold towards CMDs may be beneficial.

## Administering interventions and providing treatment

Looking towards the treatment and support healthcare providers can offer, some providers may be involved in the administration of specific interventions which can be beneficial for SRTW for those with CMDs. Arends *et al*. [30] examined the impact of the Stimulating Healthy participation And Relapse Prevention at work (SHARP-at-work) intervention, which begins within 2 weeks of an initial RTW and involves five steps. These steps include making inventories of issues and opportunities, generating solutions, writing down solutions and required support, discussing relevant information and a plan with the work supervisor, and finally evaluation of the plan and solutions. The intervention is largely administered by occupational physicians, with a minimum of two sessions with them required. The study found that those in the intervention group had lower incidence of recurrent absence and a longer median time until absence recurrence. Another intervention administered by occupational physicians was studied by van der Klink *et al*. [31]. This intervention aims to enable individuals to develop and implement problem solving strategies for regular working life issues through three stages: the first focuses on gaining insight into patient challenges and prompting patients to complete less demanding daily tasks, the second involves the identification of stressors and beneficial strategies, and the final stage focuses on putting solutions into practice and completing more demanding tasks. At least one session was completed after an individual’s initial RTW. This intervention also demonstrated a positive impact, with a lower recurrence rate observed in the intervention group. Corbière *et al*. [32] have also examined the Healthy Minds for Sustainable RTW intervention. This intervention is administered by counsellors and involves eight sessions as well as homework tasks, covering topics such as coping strategies, stress, unhelpful thinking, work functioning, work accommodations, self-esteem, handling criticism, and CMD disclosure. The intervention is conducted in a group setting, with between five and seven participants who had all recently returned to work in each group. The study found support for their hypothesis that the intervention would improve work productivity over time. Such interventions show promising results which could lead to improved SRTW outcomes, with healthcare providers playing a key role in their provision. These interventions all consist of multiple stages, with at least one session conducted after the individual has re-entered the workplace. They all also contain work-related components and have a focus on problem-solving, with individuals required to develop specific strategies for the workplace challenges they face. However, further research may wish to further assess the economic viability of interventions, with one study of the SHARP-at-work intervention finding it did not have an economic benefit compared to care-as-usual [33]. This is an important consideration if interventions are to be implemented on a larger scale.

Relatedly, a study of returnees and managers found that identifying triggering situations and functional strategies to manage challenges may support work sustainability, and this could be achieved with the support of healthcare providers [27]. Similar findings were identified through interviews by Bjørndal *et al*. [34], with treatment at an outpatient mental health clinic, including metacognitive therapy and work-focused interventions, enabling returnees to understand more about their mental health, develop new cognitive approaches, and learn various behavioral strategies for managing work. Participants noted, for example, that treatment had reduced their self-criticism and their need to feel in control, which left them with more time and a greater capacity for managing other aspects of life, including work. This suggests the healthcare provider role can involve providing suitable treatment and support to aid with CMD management and the identification of strategies to facilitate SRTW. However, Holmlund *et al*. [27] do note that access to this type of support can vary amongst workers. Smaller companies may not provide access to occupational physicians, for example [35].

## Remaining vigilant and handling future returnee challenges

Healthcare providers may also wish to pay heightened attention to groups who can be more likely to experience unfavourable outcomes following RTW. For example, a recent systematic review found that lower socioeconomic status (SES) may increase the likelihood of recurrent sickness absence due to CMDs [36]. It was noted that aspects of job design, such as decision latitude, could be related to this, as they can be worse for individuals with lower SES. The review suggested that occupational health providers may be able to contribute to the reduction of recurrent absence by providing longer-term support and offering guidance to employers, with particular focus on modifiable factors including working conditions.

As detailed earlier, ideally, returnees would be able to stay at work and thrive long after initial workplace re-entry following CMD-related absences. However, unfortunately, many individuals do appear to have challenges after returning [12, 13]. Thus, it is vital for patients to be provided with access to clinical follow-up after initial RTW to monitor their progress and identify opportunities for support. Specifically, it has been suggested that more intensive monitoring is necessary for the first 18 months after RTW [37]. Certain interventions may also be useful at this stage, with one study finding employees with depression who received work-focused counselling in addition to their integrated care programme had larger reductions in work productivity loss than those who did not receive counselling [38]. Relatedly, whilst research has often, somewhat understandably, focused on absence recurrence as an outcome of interest, healthcare providers should also pay attention to other components of SRTW, such as the returnee’s level of work functioning [11]. If a patient contacts their healthcare provider following RTW and remains in work without an absence recurrence, but struggles with wellbeing and work functioning, their case should be handled with urgency. In such scenarios, it may be that support at this stage could help to prevent future relapses. Additionally, though this review has demonstrated that healthcare providers should always prioritize taking preventative and protective actions to avoid relapses, they should also be prepared to deal with absence recurrence in their patients if it happens. It has previously been suggested that to address cases of relapse, physicians should consider referring workers to appropriate mental health professionals or rehabilitation programmes which specialize in these scenarios [39].

## Improving provider confidence and skills

Thus far, various ways that healthcare providers may be able to support SRTW have been explored. However, to ensure individual healthcare providers can make a difference, it is necessary to ensure that they have the appropriate skills, knowledge, and experience to manage RTW in patients. Unfortunately, many healthcare providers appear to have a lack of confidence around managing patient cases involving an RTW. In a study for the UK’s Department for Work and Pensions, 37% of GPs indicated that they were not confident in dealing with patient issues around RTW [7]. The same study found that over 18% of GPs surveyed did not feel their knowledge of sickness certification guidelines was up to date. A scoping review also found that GPs reported a need to improve their knowledge of absence certification processes [40]. The review also identified a need for healthcare providers to be more aware of relevant services for ill workers, such as occupational therapy support. Initiatives such as training courses from RTW specialists for individual healthcare providers could be beneficial for building provider confidence, and organizing such activities should be prioritized by healthcare senior leadership and others responsible for training and development. If there are future changes to certification processes, it is also crucial for accessible educational resources to be made available for providers. Such provisions can help to ensure that providers have the confidence and abilities to put best practices into action.

## Considerations for future research

Future research should examine whether healthcare providers view the supportive actions discussed in this review as existing within the scope of their practice, as this is currently unclear. Identifying which actions are most practical and realistic for healthcare providers to put in place would also be beneficial. Future research should also seek to evaluate the impact of any related initiatives which are implemented. For example, if healthcare providers are provided with RTW training, changes to their practices could be assessed.

Additionally, whilst this review has explored actions healthcare providers may take to improve outcomes related to the sustainability of RTW for individuals with CMDs, SRTW itself does not currently have one agreed-upon definition, as noted earlier. When providing their preliminary definition of SRTW, presented earlier, Nielsen *et al*. [11] comment that not enough is known about the factors which are important for SRTW to provide a final definition. This is an important avenue for future research to investigate, though unpacking the concept of SRTW currently raises a variety of questions. For example: can an individual’s RTW be considered sustainable even if it includes absence recurrence if the worker is performing to a level both they and their employer are pleased with? Has SRTW been achieved if working hours are consistently lower than before the absence, but there is good work functioning and no future relapse? Do all RTW outcomes need to have positive resolutions for it to be sustainable—and for how long? Providing answers to such questions, and deciphering what SRTW truly means to key stakeholders, would allow researchers to investigate the topic with increased clarity, which could ultimately further improve the understanding of how healthcare providers can facilitate SRTW.

In recent years there appears to have been an increase in research output on SRTW, though overall there has been less research focus on the sustainable phase of RTW than on other aspects of RTW. Continuing to examine how resources at all levels of the IGLOO model [11] affect SRTW can help to improve the understanding of how various actors, including healthcare providers, can improve longer-term outcomes. In particular, looking at the influence of resources at the fifth level of the IGLOO model should be a priority for future studies, as minimal research currently appears to examine this level. It could be useful, for example, to examine how national legislation affects the care given by healthcare providers, and what the resulting impact is on SRTW.

## Conclusions

Overall, evidence demonstrates that healthcare providers play a pivotal role in improving SRTW outcomes after CMD-related absences. There are multiple evidence-based actions that individual healthcare providers and healthcare systems can implement to support SRTW. These include establishing suitable RTW timeframes for their patients, prioritizing patient perspectives in decision-making, maintaining effective stakeholder coordination, contributing to mental health stigma reduction, and delivering targeted interventions and treatment for CMD management. Healthcare providers should also remain vigilant to at-risk groups and potential post-RTW challenges such as reduced work functioning and relapse. To optimize individual provider actions, healthcare leadership systems must also focus on enhancing provider confidence, knowledge, and skills in RTW management.

Moving forward, several priorities emerge for both practice and research. Healthcare providers, particularly GPs, psychiatrists, and psychologists, should recognize their unique position as leader-level resources within the broader RTW ecosystem and actively engage in the evidence-based practices outlined in this review. Future research should focus on exploring whether healthcare providers view actions identified in this review as existing within the scope of their practice, evaluating the impact of implemented initiatives, developing a refined definition of SRTW, and examining how resources across all IGLOO model levels [11] can support SRTW. Through these combined efforts of practice enhancement and continued research, healthcare providers can better fulfil their role in supporting SRTW for individuals with CMDs.

## Data Availability

As this is a review paper, no new empirical data were created or analyzed, and data sharing is not applicable.

## Copyright licence

For the purpose of open access, the author has applied a Creative Commons Attribution (CC BY) licence to any Author Accepted Manuscript version arising from this submission.
